# Efficacy and Renal Safety of Dapagliflozin in Patients with Type 2 Diabetes Mellitus Also Receiving Metformin: A Real-Life Experience

**DOI:** 10.1155/2018/8501418

**Published:** 2018-05-03

**Authors:** Alessandro Scorsone, Gabriella Saura, Mattia Fleres, Lucia Spano, Vito Aiello, Davide Brancato, Anna Di Noto, Francesca Provenzano, Vincenzo Provenzano

**Affiliations:** ^1^Regional Referral Centre for Insulin Pump Implantation and Diabetes, Civic Hospital, Partinico, Palermo, Italy; ^2^Department of Internal Medicine, University of Palermo, Palermo, Italy

## Abstract

**Introduction:**

This study aimed at evaluating the efficacy and safety of dapagliflozin in patients with type 2 diabetes (T2D) who also received metformin in clinical practice in Italy.

**Methods:**

This was a retrospective observational study and it included data from patients who received dapagliflozin 10 mg once daily in conjunction with metformin for 12 months (DAPA + MET). In those with inadequate glycemic control, insulin or glimepiride was added after 30 days (DAPA + MET + other glucose-lowering drugs). Efficacy assessments included glycosylated hemoglobin (HbA_1c_) levels at 6 and 12 months, as well as body mass index (BMI) and lipid parameters at 12 months. Safety was also assessed.

**Results:**

Data on 66 patients were included. In both groups, HbA_1c_ was significantly reduced at 6 and 12 months compared with baseline and significant reductions in HbA_1c_ were observed at 12 months compared with 6 months. Over the 12-month treatment period, dapagliflozin significantly reduced BMI in both groups. No significant changes in lipid parameters were observed in either group and no detrimental effects on renal function were detected.

**Conclusions:**

Dapagliflozin is effective and safe in patients with T2D also receiving metformin. Glycemic control was already achieved with dapagliflozin + metformin, and add-on therapy was not associated with further improvements.

## 1. Introduction

Over the past 20 years, the proportion of Italians with diabetes in the general population increased from 3.4% to 5.5% and the vast majority (91%) have type 2 diabetes. In Italy, the management of diabetes is associated with approximately €10 billion per year in direct and indirect costs [1].

The principal goal of effective treatment of type 2 diabetes is to reduce blood glucose [2]. At the same time, because type 2 diabetes is characterized by systemic dysregulation of metabolism and is strongly associated with obesity [3], glucose-lowering agents that reduce body weight are preferable to those that have no effect on or increase it. Cardiovascular diseases, for which obesity is a major risk factor, are estimated to cause 40% of all deaths attributed to type 2 diabetes [4].

Dapagliflozin is a glucose-lowering agent that acts by inhibiting sodium glucose cotransporter 2 (SGLT2). Located in the proximal tubule of the nephron, SGLT2 is responsible for the reabsorption of most of the previously filtered glucose [5]. SGLT2 inhibition results in glycosuria and represents an insulin-independent method of reducing blood glucose levels. It also results in a reduction in body weight due to the loss of calories contained in excreted glucose [6]. Studies have demonstrated that dapagliflozin reduced serum glycosylated hemoglobin (HbA_1c_) levels and body weight in patients with type 2 diabetes when used as monotherapy [7], as well as in combination with other glucose-lowering agents [8–11].

The aim of this study was to evaluate the efficacy and safety of dapagliflozin in patients with type 2 diabetes also receiving metformin in clinical practice in Italy.

## 2. Methods

### 2.1. Study Design

This retrospective observational study investigated the effectiveness and renal safety of dapagliflozin 10 mg once daily in adults with type 2 diabetes mellitus who were also receiving metformin 1.5–2.5 g/day (DAPA + MET). Data from outpatients treated at the Regional Referral Centre for Insulin Pump Implantation and Diabetes at the Civic Hospital of Palermo, Italy, who initiated dapagliflozin treatment between March 2015 and March 2016 and who had undergone a follow-up visit at 12 months were included in the analysis. We included 66/94 subjects with type 2 diabetes inadequately controlled with metformin who attended our outpatient clinic for the entire period of observation, had never discontinued therapy, had never had side or adverse effects, had normal renal function (estimated glomerular filtration rate over 60 mL/min), modified their dietary habits according to our advices, and since baseline, underwent all blood tests in our outpatient clinic laboratory. If they were not able to perform self-blood glucose testing at home or did not modify according to our advices, then they were excluded. In patients whose HbA_1c_ level was ≥7.5%, insulin or glimepiride were added to the treatment regimen at the same time of initiation of dapagliflozin (DAPA + MET + other glucose-lowering drugs), as per normal clinical practice and clinician's decision.

The ethics committee (Comitato Bioetico Palermo) at the investigational site was notified of the study protocol and the study was conducted in accordance with the Italian law and the Declaration of Helsinki.

### 2.2. Assessments

Baseline characteristics included age, sex, weight, waist circumference, systolic blood pressure, diastolic blood pressure, and disease duration.

Effectiveness was assessed using the change from baseline in HbA_1c_ at 6 and 12 months as well as changes in body mass index (BMI) and lipid parameters (total cholesterol, low density lipoprotein (LDL) cholesterol, high density lipoprotein (HDL) cholesterol, and triglycerides) at 12 months. HbA_1c_ was self-monitored, with outpatient visits performed every three months or when needed. Renal function was assessed by the change in blood creatinine levels at 6 and 12 months, as well as urine microalbumin levels at 12 months.

### 2.3. Statistical Analyses

Statistical analyses were performed using SPSS Statistics version 21 for Windows. All variables were analyzed by summary statistical methods and the analyses were performed separately in patients who received DAPA + MET and DAPA + MET + other glucose-lowering drugs. For continuous/quantitative variables, descriptive statistics, including the number of available values, arithmetic mean, standard deviation (SD), minimum, median, and maximum were calculated, while for categorical/qualitative variables, frequency tables were generated. Paired sample *t*-tests were performed using an analysis of covariance (ANCOVA) model with baseline values as a covariate with a significance level set at 5%. General linear models were used to adjust for variation between patients who received DAPA + MET and those who received DAPA + MET + other glucose-lowering drugs. Baseline values, age, and disease duration served as covariates, while sex served as the fixed factor.

## 3. Results

Data on 66 patients (mean age 56 years; 39% female) were included in the study after database analysis of 235 type 2 diabetic subjects as reported in the Methods. At baseline, mean HbA_1c_ of the entire patient population was 9.2%, while mean eGFR was 95.5 mL/min. Concomitant therapies included angiotensin-converting-enzyme inhibitors, angiotensin receptor blockers and calcium channel blockers for hypertension, and statins for management of dyslipidemia. The baseline demographics and characteristics were not significantly different between patients who received DAPA + MET alone and those who were prescribed additional therapy (DAPA + MET + other glucose-lowering drugs), with the exception of waist circumference and disease duration (Table 1). After 12 months of therapy, 29 (44%) patients had initiated treatment with an additional glucose-lowering agent (glimepiride 2–4 mg/day or insulin) and 12 (18%) patients had discontinued metformin due to intolerance.

### 3.1. Effectiveness

**Figure 1:**
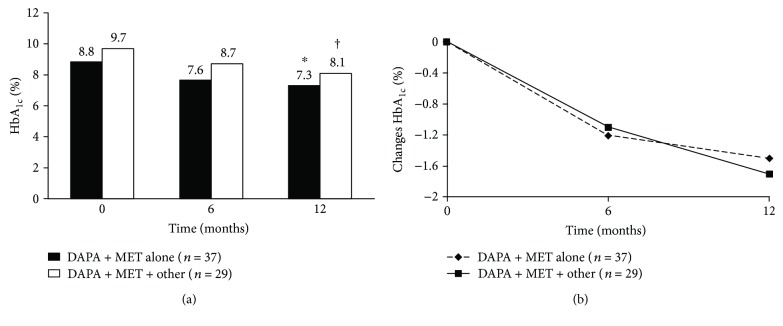
Glycosylated hemoglobin (HbA_1c_) (a) at each study visit and (b) change from baseline. DAPA, dapagliflozin; MET, metformin; other, glimepiride or insulin. ^∗^*p* < 0.001 DAPA + MET versus baseline; ^†^*p* < 0.001 DAPA + MET + other glucose-lowering drugs versus baseline.

When added to metformin, dapagliflozin significantly reduced HbA_1c_ levels from baseline in patients who received DAPA + MET, as well as in those who received DAPA + MET + other glucose-lowering drugs (Figure 1). Significant reductions were observed after 6 and 12 months of treatment. Moreover, in all patients significant reductions were observed at 12 months compared with the HbA_1c_ levels at 6 months (Figure 1(b)). After 6 months of treatment, HbA_1c_ levels decreased by 1.4% (95% confidence interval (CI): 1.1% to 1.7%; *p* < 0.001) and 0.8% (95% CI: 0.5% to 1.1%; *p* < 0.001) in patients who received DAPA + MET and those who also received other glucose-lowering drugs, respectively. After 12 months of treatment, HbA_1c_ levels decreased by 1.7% (95% CI: 1.5% to 2.0%; *p* < 0.001) and 1.3% (95% CI 1.0% to 1.6%; *p* < 0.001), respectively. In patients who received DAPA + MET, the change from baseline in HbA_1c_ levels at 6 months was significantly greater that in patients who received DAPA + MET + other glucose-lowering drugs (*p* = 0.020); however, no significant between-group difference was observed at 12 months (*p* = 0.053). Over the 12-month treatment period, dapagliflozin significantly reduced BMI in patients who received DAPA + MET (ΔBMI = −0.7 kg/m^2^, 95% CI: −1.1 to −0.3; *p* < 0.01) and DAPA + MET + other glucose-lowering drugs (ΔBMI = −0.9 kg/m^2^, 95% CI: −1.3 to −0.5, *p* < 0.001) (Table 2), with no significant difference between patients. No significant changes in lipid parameters over the study period were observed.

### 3.2. Renal Function

**Figure 2:**
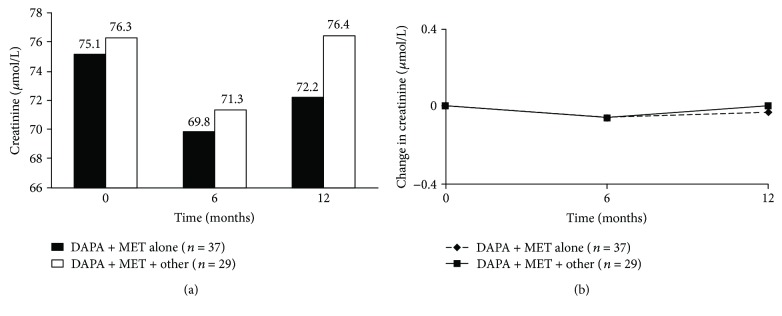
Creatinine (a) at each study visit and (b) change from baseline. DAPA, dapagliflozin; MET, metformin; other, glimepiride or insulin.

No detrimental effects on renal function were observed during the study. There were no significant changes from baseline in creatinine levels at 6 and 12 months in all patients (Figure 2). On the other hand, after 12 months, a significant reduction in microalbumin was observed in patients who received DAPA + MET alone (Table 2). The eGFR values in all patients remained above 60 mL/min throughout the study, in accordance with the prescribing rules of the Italian Medicines Agency (AIFA). No statistically significant variation in eGFR was observed between treatment groups.

## 4. Conclusions

This retrospective observational study investigated the effectiveness and safety of add-on therapy with dapagliflozin in patients with type 2 diabetes treated at a single facility in Italy. The results demonstrate that dapagliflozin is effective and safe. Dapagliflozin significantly reduced HbA_1c_ levels after 12 months of treatment regardless of whether other glucose-lowering agents were received. In both patients, significant reductions were apparent from 6 months. Contrary to expectations, greater reductions were obtained in patients who received DAPA + MET, that is, those who received no additional therapy. We did not find any negative effects on renal function (assessed by blood creatinine and urine microalbumin levels) during the study. Microalbumin reduction was observed in patients who received DAPA + MET alone but we cannot exclude, due the nature of our study, other potential factors that could explain this exclusive effect.

The results of this study are in line with those of other studies of dapagliflozin conducted in patients with type 2 diabetes inadequately controlled with metformin [8–16].

This study had a number of limitations due to its retrospective observational design. It used data from an unselected group of patients and the size of the patient population was not as large as in other studies conducted in patients with type 2 diabetes. Furthermore, no measures were taken to control for confounding factors. This, in combination with a lack of a control group, means that this study cannot adequately explain the finding that patients treated with DAPA + MET experienced greater reductions in HbA1c levels compared with those who received additional glucose-lowering drugs. The strengths of this study, on the other hand, included the length of the observational period and the fact that it was conducted in a real-life setting. These factors improve the generalizability of its findings. In conclusion, this retrospective observational study demonstrates that dapagliflozin is effective in reducing HbA_1c_ levels and safe when administered in a real-life setting in Italy in patients with type 2 diabetes also receiving metformin. Glycemic control was more likely to be achieved in patients treated with dapagliflozin alone or in combination with metformin, while add-on therapy with insulin and/or glimepiride was not associated with further improvement in HbA_1c_ levels.

## Figures and Tables

**Table 1:** Baseline demographics and characteristics.

Characteristic	DAPA + MET alone (*n* = 37)	DAPA + MET + other glucose-lowering drugs (*n* = 29)^a^	Total (*n* = 66)
Age (years)	57.1 ± 9.3	55.1 ± 8.6	56.3 ± 9.0
Female, *n* (%)	14 (37.8)	12 (41.4)	26 (39.4)
Weight (kg)	91.6 ± 17.9	94.6 ± 17.6	93.0 ± 17.7
Waist circumference (cm)	111.0 ± 15.0	119.0 ± 16.2^∗^	114.5 ± 15.9
Disease duration (years)	8.1 ± 5.0	11.6 ± 8.5^∗^	9.7 ± 6.9
Systolic BP (mmHg)	138.6 ± 18.5	134.7 ± 14.0	136.8 ± 16.6
Diastolic BP (mmHg)	81.5 ± 9.3	81.0 ± 12.6	81.3 ± 10.8
HbA_1c_ (%)	8.8 ± 1.6	9.7 ± 1.5	9.2 ± 1.6
BMI (kg/m^2^)	33.1 ± 6.2	33.8 ± 6.3	33.4 ± 6.1
Creatinine (*μ*mol/L)	75.1 ± 17.6	76.3 ± 15.5	75.6 ± 18.2
Microalbumin (*μ*g/mg)	119.5 ± 139.6	67.1 ± 80.6	96.5 ± 118.6
eGFR (mL/min)	95.9 ± 29.1	96.6 ± 24.1	95.5 ± 29.7

All values are presented as mean ± standard deviation unless otherwise stated. BMI, body mass index; BP, blood pressure; DAPA, dapagliflozin; eGFR, estimated glomerular filtration rate; HbA_1c_, glycosylated hemoglobin; MET, metformin. ^a^Additional glucose-lowering agents included glimepiride or insulin. ^∗^*p* < 0.05 versus DAPA + MET alone.

**Table 2:** Secondary efficacy and safety parameters.

	DAPA + MET alone (*n* = 37)		DAPA + MET + other glucose-lowering drugs (*n* = 29)^a^	
	Baseline	12 months	Baseline	12 months
BMI (kg/m^2^)				
Mean ± SD	33.1 ± 6.2	32.5 ± 6.0	33.8 ± 6.3	32.9 ± 5.2
Δ	−0.7 (−1.1 to −0.3)^∗∗^		−0.9 (−1.3 to −0.5)^∗^	
*p* Value	<0.001		0.009	
Total cholesterol (mg/dL)				
Mean ± SD	189.5 ± 33.9	184.1 ± 40.1	183.8 ± 42.2	177.9 ± 32.1
Δ	−2.2 (−15.8 to +11.4)		−10.9 (−25.2 to +3.4)	
LDL cholesterol (mg/dL)				
Mean ± SD	111.1 ± 32.2	104.9 ± 36.4	108.1 ± 38.2	103.6 ± 31.6
Δ	−4.8 (−17.0 to +7.4)		−9.5 (−22.4 to +3.3)	
HDL cholesterol (mg/dL)				
Mean ± SD	47.7 ± 11.9	52.2 ± 13.0	48.5 ± 11.8	48.3 ± 10.6
Δ	+4.8 (+0.6 to +9.1)		+0.8 (−3.8 to +5.3)	
Triglycerides (mg/dL)				
Mean ± SD	145.8 ± 62.9	135.1 ± 54.2	134.8 ± 61.2	129.7 ± 57.2
Δ	−4.5 (−24.6 to +15.6)		−5.8 (−27.0 to +15.5)	
Microalbumin (*μ*g/mg)				
Mean ± SD	119.5 ± 139.6	93.6 ± 103.2	67.1 ± 80.6	54.0 ± 52.3
Δ	−22.9 (−30.6 to −15.2)^∗^		−18.6 (−27.1 to −10.0)	
*p* Value	0.002		0.070	

Δ = mean (95% confidence interval) change from baseline to 12 months. BMI, body mass index; DAPA, dapagliflozin; HDL, high density lipoprotein; LDL, low density lipoprotein; MET, metformin; SD, standard deviation. ^a^Additional glucose-lowering agents included glimepiride or insulin. ^∗^*p* < 0.001 and ^∗∗^*p* < 0.01.
